# Proceedings of Societies

**Published:** 1868-06-15

**Authors:** 


### PROCEEDINGS OF THE PEORIA COUNTY MEDICAL SOCIETY.

A MEETING of the Society was held at Princeville, May 15th. The President, Dr. G. L. Corcoran, in the chair.

Dr. R. F. Henry was chosen Secretary *pro tem.*

The minutes of the previous meeting were approved as reported by Dr. J. K. Secord.

On motion, Drs. Allen, Thomas, and Wilmot, of Chillicothe; Charles, Andrews, Emery, and Henry, of Princeville; and Lane, of Tivoli, were elected members of the Society.

Drs. H. Steele and G. L. Corcoran were appointed delegates to the Illinois State Medical Society, to meet at Quincy on Tuesday next; Drs. R. F. Henry and G. W. Emery, Alternates.

The subjects proposed for discussion at the next meeting — Uræmia, and Type Fevers.

The Sectetary was instructed to furnish a copy of the proceedings of this meeting to the CHICAGO MEDICAL JOURNAL, and the Peoria and Elmwood papers, for publication.

On motion of Dr. Steele, the Society adjourned, to meet at Elmwood on the second Wednesday of July next, at 1 oclock, P.M.

PRINCEVILLE, ILL., *May* 15, 1868. R. F. HENRY, *Sec'y.*

---

### THE LATE DR. R. T. RICHARDS.

AT the annual meeting of the DeWitt County Medical Society, held on the 11th day of May, 1868, Dr. C. Goodrake, from the committee appointed to draft resolutions expressive of the sense of this Society on the death of our late colleague, Dr. Richards, reported the following preamble and resolutions, which were unanimously adopted:

*Whereas*, It has pleased God, in His wise Providence, to remove from our midst, and from a field of great professional usefulness, our highly esteemed friend and co-laborer in the science and practice of medicine, Dr. ROLLA T. RICHARDS, who died at Santa Anna, on the 12th day of March, 1868; be it therefore

*Resolved*, That we deeply feel the loss of our deceased friend and brother, who, by his upright, moral conduct, and gentlemanly deportment, had endeared himself to all with whom he became associated.

*Resolved*, That in his death, the profession is deprived of one of its most zealous and devoted members, and the community of a *good physician*, and one of her best and most patriotic citizens.

*Resolved*, That we feel a sincere sympathy for his widow in her great affliction, and can only refer her, with unfeigned confidence, to Him who has promised to be the widow's God.

*Resolved*, That the foregoing preamble and resolutions be spread upon the records of the Society; an attested copy sent to the widow of the deceased, and one to each of the medical journals published in Chicago, for publication.

C. GOODBRAKE,<br>

J. WRIGHT, } *Committee.*<br>

Z. H. MADDEN,

J. A. EDMISTON, M.D., *President.*

C. GOODBRAKE, M.D. *Secretary.*

